# Mining and analysis of audiology data to find significant factors associated with tinnitus masker

**DOI:** 10.1186/2193-1801-2-595

**Published:** 2013-11-08

**Authors:** Muhammad Naveed Anwar

**Affiliations:** The Open University, Milton Keynes, UK

**Keywords:** Tinnitus, Masker, Audiogram, Age, Gender, ITE (in the ear)/BTE (behind the ear) hearing aids, Mould

## Abstract

**Objectives:**

The objective of this research is to find the factors associated with tinnitus masker from the literature, and by using the large amount of audiology data available from a large NHS (National Health Services, UK) hearing aid clinic. The factors evaluated were hearing impairment, age, gender, hearing aid type, mould and clinical comments.

**Design:**

The research includes literature survey for factors associated with tinnitus masker, and performs the analysis of audiology data using statistical and data mining techniques.

**Setting:**

This research uses a large audiology data but it also faced the problem of limited data for tinnitus.

**Participants:**

It uses 1,316 records for tinnitus and other diagnoses, and 10,437 records of clinical comments from a hearing aid clinic.

**Primary and secondary outcome measures:**

The research is looking for variables associated with tinnitus masker, and in future, these variables can be combined into a single model to develop a decision support system to predict about tinnitus masker for a patient.

**Results:**

The results demonstrated that tinnitus maskers are more likely to be fit to individuals with milder forms of hearing loss, and the factors age, gender, type of hearing aid and mould were all found significantly associated with tinnitus masker. In particular, those patients having Age < =55 years were more likely to wear a tinnitus masker, as well as those with milder forms of hearing loss. ITE (in the ear) hearing aids were also found associated with tinnitus masker. A feedback on the results of association of mould with tinnitus masker from a professional audiologist of a large NHS (National Health Services, UK) was also taken to better understand them. The results were obtained with different accuracy for different techniques. For example, the chi-squared test results were obtained with 95% accuracy, for Support and Confidence only those results were retained which had more than 1% Support and 80% Confidence.

**Conclusions:**

The variables audiograms, age, gender, hearing aid type and mould were found associated with the choice of tinnitus masker in the literature and by using statistical and data mining techniques. The further work in this research would lead to the development of a decision support system for tinnitus masker with an explanation that how that decision was obtained.

## Background and significance

This research deals with a big heterogeneous audiology data from a large NHS (National Health Services) facility, containing audiograms, structured data (such as age, gender, diagnosis) and free text (containing specific information about each patient, i.e., clinical comments). This research looks for factors associated with tinnitus masker on the basis of large data by doing the mining and analysis of this data.

Deafness Research UK (2010) states that nearly five million people in the UK live with tinnitus. Some estimates show that 40 million people in the United States may be affected by tinnitus and 10 million of them consider their problem significant (Folmer 2002). In many cases the exact reason for tinnitus is not known and diagnosis typically begins with a visit to an ear specialist. A detailed clinical evaluation, including a thorough patient history and physical check-up of the head and neck together with the different nerves in that region, and audiometric testing are performed by the doctor for the diagnosis of tinnitus and its causes. For example, ear wax, ear infection, and vestibulocochlear nerve (auditory nerve) that controls the sense of hearing and balance are checked. A detailed audiogram is also taken. Some tinnitus may be caused by neurologic, metabolic, or psychogenic disorders (Crummer and Hassan 2004). Depending on the type of tinnitus, either a special audiogram known as an auditory brainstem response (ABR) (a test that gives information about the inner ear (cochlea) and brain pathways for hearing) or a brain scan such as a magnetic resonance imaging (MRI) scan or computerised tomography (CT) may also be taken. In a few cases, blood pressure and possibly blood tests for hyperthyroidism (a condition that the thyroid gland makes too much thyroid hormone) are also performed. In a very few cases, a spinal tap procedure is used to find the fluid pressure in the skull and spinal cord. In the section below, some of the causes and problems associated with tinnitus and its treatment found in previous audiology research are described.

### Tinnitus causes

Tinnitus can be caused by different factors including exposure to loud noises, head trauma, and a variety of diseases (Snow 2004). Tinnitus can be induced in 94% of the population by a few minutes of sound deprivation (Heller and Bergman 1953). Tinnitus can cause severe emotional distress to patients (Andersson and McKenna 1998), and results in disturbing the life of a person. Below are some of the causes of tinnitus found in the audiology literature:

#### Hearing impairment

Tinnitus is found to be highly correlated with hearing impairment and severity of impairment (Davis 1989). It has also been found that high frequency hearing loss is a predictor of tinnitus (Jakes and Stephens 1987). In the given audiology data, it was found that patients with mild to moderate hearing loss group were more likely to have tinnitus and be using a masker, and also in this group patients were more concerned with tinnitus than the hearing loss.

#### Age

The likelihood of having tinnitus increases with age and about 30% of the population affected with tinnitus have been found to be over 55 years of age (Lockwood et al. 1998). In a previous research on a large audiology data, it was found that patients having age less than or equal to 54 (i.e., *Age* ≤ 54) were less likely to have tinnitus (Anwar et al. 2011).

#### Gender

It is reported that women with tinnitus seem to report more psychological distress, though the exact cause of this gender difference is not well understood (Dineen et al. 1997). In a study on tinnitus and hearing loss in Sweden, Brunnberg et al. (2008) found a gender difference in hard-of-hearing 15–16-year-old young students, but they did not find the gender difference in students with normal-hearing and having tinnitus. In a previous study on a large audiology data, there was no association found of gender with tinnitus (Anwar et al. 2011). Miekle and Griest (1989) found that more male tinnitus sufferers had histories of noise exposure than women, and that women have a greater tendency to report tinnitus on both sides (as opposed to in one ear only) than men.

### Tinnitus treatments and problems associated with them

Jastreboff and Hazell (2004) described tinnitus treatment a promising, expensive and complex that may span from several months to a couple of years. It is described in the literature that there is neither any cure for tinnitus (Vio and Holme 2005; Gander et al. 2011) nor any licensed medications for minimising its symptoms (Vio and Holme 2005). For example, there is no Food and Drug Administration (FDA) or European approved drug specifically for treating subjective tinnitus (Langguth et al. 2009). The reason for this is the heterogeneity observed in the tinnitus population (Moller 1997; Landgrebe et al. 2010), so there is no single theory, model or hypothesis that explains the presence of tinnitus in all those affected (Baguley and McFerran 2002). Gander et al. (2011) mentioned that there are no comprehensive surveys about tinnitus referral systems from the clinician’s perspective. Thus, most of the tinnitus treatment options are mainly for lessening or managing the existing symptoms of tinnitus by making them less distressing (Jastreboff and Hazell 2004; Vio and Holme 2005). Landgrebe et al. (2010) mentioned that one of the major problems of tinnitus research is that it is a subjective experience (like pain) and cannot be measured by using objective measurement methods. For these reasons, it is difficult to predict who will suffer from tinnitus (Tyler and Stouffer 1989). Tinnitus can only be quantified by using self-rating questionnaires. It is noted that an important factor for effective management of tinnitus patients is a quick process of triage and referral to an appropriate professional (Gander et al. 2011).

The fitting of masking devices plays an important part in the management of the many different forms of tinnitus (Hazell et al. 1985; Henry et al. 2004). Tinnitus maskers are small devices worn like hearing aids. They give relief from tinnitus by giving out a “white noise” or an ultra-sonic signal to cover up the internal sounds heard. Tinnitus masking has been in use as a clinical technique since 1976 (Vernon 1976). There are no harmful effects of masking on hearing (Hazell et al. 1985). The other treatments that are useful in the management of tinnitus are as follows:

- Hearing aids are found to give masking relief for about 12% of tinnitus patients (Vernon 1988). But in a recent study it was found that tinnitus patients with hearing aids report slightly fewer benefits and more problems with their hearing aids particularly for background sounds and intolerance of sounds (Andersson et al. 2011).
- On some occasions, when none of the other treatments is effective for tinnitus, surgery is advised to produce relief from severe effects of tinnitus. These cases are because of injuries in the inner ear and are generally related with severe or total deafness. These surgical procedures require a high degree of technical skill.
- TRT (Tinnitus Retraining Therapy) is another method of tinnitus treatment (Jastreboff and Jastreboff 2000). Its primary objective is to facilitate habituation to tinnitus (Henry et al. 2002; 2004). This method was first described in 1990, and is based on a neurophysiological model of tinnitus (Jastreboff 1990). It combines medical evaluation, counselling and sound therapy for the management of tinnitus to help the majority of tinnitus patients (Zhang et al. 2011).
- Tinnitus counselling is designed to enable the understanding of tinnitus to deal with it efficiently. It is usually conducted by hearing therapists. It is a discussion therapy that helps the patient to understand in detail about tinnitus and the methods to cope with it. Discussing tinnitus makes one understand more about it and possibly lessens its effect.
- Cognitive behavioural therapy (CBT) is related to many therapies that aim to deal with issues such as nervousness, sadness and post-traumatic stress disorder (PTSD). In the daily lives of people emotions affect their behaviour, and this is the basis of CBT. For example, a patient having a lack of information about tinnitus might have ideas that would make him more nervous and upset. But in reality these ideas are false and giving the correct information about tinnitus might help to improve the condition.
- Self-help techniques, such as taking rest, listening to music and joining tinnitus support groups, are also important for people for dealing with their tinnitus. For example, anxiety can increase tinnitus, therefore doing exercise regularly such as meditation or yoga might reduce tinnitus. Joining a tinnitus support group also gives the opportunity to share tinnitus experience with other tinnitus sufferers, which can also prove helpful by giving more understanding of tinnitus and how to better deal with it.

The minimal masking level (MML) of tinnitus is a possible predictor of acceptance of tinnitus masker treatment and also a measure of treatment success (Vernon et al. 1990; Jastreboff et al. 1994). The Beck Depression Inventory (BDI) is also used as a measure of depression (Beck et al. 1961). It uses a self-report instrument to measure the level of depression in tinnitus patients (Andersson and McKenna 1998; Kirsch et al. 1989). The higher the total scores of the responses, the greater the signs of depression.

### Tinnitus maskers

One interesting and strange distinction among tinnitus patients regarding the effect of background noise for treating the tinnitus is that noise makes tinnitus worse for some patients but reduces tinnitus for others (Stouffer and Tyler 1990; Tyler and Baker 1983). It is important to provide careful counselling and follow up in order to obtain optimal results, when fitting a device, masker, aid or combination instrument (Hazell and Wood 1981). Maskers are often more effective than hearing aids (Hazell et al. 1985), though hearing aids have been reported to provide masking relief for about 12% of tinnitus clinic patients (Vernon 1988). Opinion about the effectiveness of maskers in the management of tinnitus varies as 71-88% of masker users and 53-68% of hearing aid users found their instrument helpful when tinnitus was troublesome (Coles and Hallam 1987). A study on a group of tinnitus patients found that in the long term 45% of tinnitus patients used maskers with or without hearing aids, and 35% used hearing aids alone (Stephens and Corcoran 1985). Another study performed initial investigations on the effectiveness of various sounds which were composed to provide tinnitus relief as compared to broadband or spectrally shaped noise, the two bands of noise commonly used in clinics, and it was found that those specially designed sounds were more effective than the typically used sounds (Henry et al. 2004). Masking for speech tests commonly uses white noise (actually broadband noise), pink noise, speech-shaped noise, or multitalker babble (Gelfand 2009). Broadband noise contains a wide range of frequencies, i.e. upwards of several octaves. White noise is a broadband noise which offers equal energy in all frequencies. Pink noise features less energy in the higher frequencies (above 8000 Hz) and is the noise that most mimic day to day living. Speech shaped noise has a spectrum that approximates of the long-term spectrum of speech. Multitalker babble is made by recording the voices of many people who are talking simultaneously, resulting in an unintelligible babble.

The choice of different masking devices in various studies is rarely specified as this is often based on their local availability and the clinician’s link with the different manufacturers rather than on any scientific appraisal (Hazell et al. 1985). There are two types of masking: continuous (subdivided into complete and partial masking) and inhibitory. In continuous complete masking, the noise “covers up” the tinnitus so that it is not audible while the masker is in use (Coles and Hallam 1987; Newman and Sandridge 2006). Its applicability depends on the noise having a more acceptable quality then the tinnitus. The complete masking phase is only recommended for patients requiring immediate relief (Newman and Sandridge 2006). For all other patients, partial masking is the recommended initial phase for sound therapy. In partial masking, a masker is used to deliver such a low-level background sound at level a slightly below the tinnitus, which facilitates tinnitus habituation (Newman and Sandridge 2006). Inhibitory masking is an alternative to continuous masking, as its frequency-specificity is much greater than for continuous masking (Coles and Hallam 1987). In this research, the audiology database has the records of different types of maskers prescribed to patients, and it was found that patients classified in moderate to severe hearing loss group were less likely to have tinnitus masker which is described later in the section of “Clusters of audiology data for tinnitus masker”.

### Problems with Tinnitus studies–small samples of tinnitus data

Landgrebe et al. (2010) mentioned that many clinical studies of tinnitus have used small patient samples, which makes it difficult to find the forecasters of treatment response for the various methods. They further stated that there is limited inter-study comparison due to various procedures used for the assessment of tinnitus and their varying outcome elements. Because of these reasons there is a need for a global repository of tinnitus patients who have undertaken certain treatments, and are assessed during the course of this treatment with standardised instruments such as, psychoacoustic measures and questionnaires. This repository has been constructed by the Tinnitus Research Initiative (TRI) database project (Tinnitus Research Initiative, 2013), which is also discussed in the paper by Landgrebe et al. (2010). The main purposes of this database are: to collect a standardised set of data on patient characteristics, treatments, and outcomes from tinnitus patients consulting specialized tinnitus clinics all over the world (currently 19 centres in 11 countries), outlining various subtypes of tinnitus from the data that has been collected at these centres, and discovering forecasters for individual treatment response based on the clinical profile. This database started in 2008 and now it contains the data of almost 3000 patients. It is anticipated that the number of centres will increase and this will grow the patient data quickly, so that this global database will help the research in future and will add to the expansion of patients’ treatment on the basis of facts.

Another repository, which in fact was the first attempt at systematically collecting tinnitus patient related data and making it freely available was the web-based Oregon Tinnitus Data Archive (Meikle 1997) http://www.tinnitusarchive.org/ (Accessed 7 November 2013). This database contains a statistical overview of past and present status of patients’ tinnitus data (such as patients’ age as a frequency histogram, comparison of the mean audiograms for different groups of patients), various outcomes of tinnitus examinations (such as, sound frequency equal to the perceived pitch of a patient’s tinnitus), audiological examinations (such as, hearing levels), and demographic information for 1630 patients with tinnitus who were examined at Tinnitus Clinic of the Oregon Hearing Research Center, Department of Otolaryngology, Oregon Health & Science University, between 1981 to 1994. This database was created for classifying and preparing different studies of tinnitus causes, for research on the tinnitus affected population such as assessing and managing tinnitus. The existence of this database has contributed much towards the understanding of tinnitus (Folmer and Griest 2003) but there are limitations to it (Landgrebe et al. 2010), such as it contains only cross-sectional data, i.e., does not inform about the relation between specific tinnitus features and the outcomes of specific treatment methods for tinnitus. This database has highly selected patient data and does not contain any validated measures for the severity of tinnitus. This database was last updated in 1994.

This research is based on a large data from a hearing aid clinic. It contains 180,000 individual records covering more than 23,000 different patients. This data is unique in that it has both types of hearing aids that is BTE (behind the ear) and ITE (in the ear) prescribed to patients as ITE hearing aids are generally not available on NHS, UK. The factors found in the literature for tinnitus masker are audiograms, age, gender and hearing aid type. These factors along with other factors–mould and comment texts, are also available in the available audiology data. All the available records in the database for each field (factors mentioned above) under study were used for tinnitus with/without masker, keeping the criterion that none of the field values should be empty. Although, this research uses a large audiology data but it also faced the problem of limited data for tinnitus, as there were only 1,316 records for tinnitus and other diagnosis.

## Objective

The objective of this research is to find the factors associated with tinnitus masker from the literature, and by using the large amount of audiology data available from a large NHS, UK hearing aid clinic.

## Materials and methods

This large audiology data contains two types of data about tinnitus that is, one of the diagnosis types given in the diagnosis field is “tinnitus”, and there is also a “masker” field where the type of tinnitus masker prescribed (if any) is recorded, in this study for processing the data for masker field only the presence or absence of masker is considered. It is important to mention that this audiology data was anonymised as there were NO actual patient ids, names, addresses and phone numbers given. Thus, no actual patient can be traced down.

### Clusters of audiology data for tinnitus masker

The background and significance section discussed a number of causes and problems related to tinnitus and maskers. Tinnitus causes such as hearing impairment, age and gender have been found in the literature and these factors will also be used in this study. The most common treatment for tinnitus is the use of a masker. Since this large database includes both which patients were diagnosed with tinnitus and which were fitted with a tinnitus masker, these fields can act as the gold standard for finding significant variables associated with tinnitus masker.

Clustering of hearing aid patient audiograms was performed on right ear air conduction (ac) frequencies from 250 to 8000 Hz (given in Tables 1 and 2) by using the k-means algorithm on 10,437 and 1,316 records with comments text and diagnosis (tinnitus and Others) respectively. The two main clusters were found for two sets of records, those for which the diagnosis field was filled in, and those for which the comment text field was filled. In each case the class exemplar (cluster centroid) of each cluster, being the mean of the audiograms contained within each cluster, was calculated (including only tinnitus diagnosis), as shown in Tables 1 and 2. In Table 1, cluster 1 (or C1) corresponds to “moderate to severe hearing loss” and cluster 2 (or C2) corresponds to “normal or near-normal hearing” for tinnitus with or without a masker. In Table 2, the class exemplars show that cluster 1 (or C1) consists of patients with “severe hearing loss” and cluster 2 (or C2) consists of patients with a “mild to moderate hearing loss” for comment text/clinical comments.

**Table 1:** **Class exemplars of each cluster of right ear air conduction frequencies for records recording the presence of tinnitus with or without a masker**

	ac250	ac500	ac1K	ac2K	ac4K	ac8K
C1	62.34	62.57	63.55	67.34	77.57	83.50
C2	21.17	17.66	17.09	20.27	34.57	42.50

**Table 2:** **Class exemplars of each cluster of right ear air conduction frequencies for comments text**

	ac250	ac500	ac1K	ac2K	ac4K	ac8K
C1	73.66	73.00	74.99	80.48	91.08	108.21
C2	35.17	33.56	35.87	43.26	55.90	66.50

The Chi-squared test is a simple way to provide estimates of quantities of interest and related confidence intervals (Altman 1991). It is a measure of associations between variables (such as the fields of the tables in a relational database) where the variables are nominal and related to each other (Lucy 2005). The Chi-squared test is popular in the medical domain because of its simplicity. It has been used in pharmacology to classify text according to subtopics (Oakes et al. 2001). The resulting chi-squared value is a measure of the differences between a set of observed and expected frequencies within a population, and is given by the formula:1

where r is the number of unique terms in a particular field of the patient records such as Tinnitus with/without masker, corresponding to rows in Table 3. c is the number of categories in the data (such as clusters) corresponding to columns in Table 3.

**Table 3:** **Observed and expected frequencies for right ear tinnitus with/without masker**

Tinnitus	Cluster 1	Cluster 2	Row total
With masker	40	391	431
	(65.58)	(365.42)	
	[654.27]	[654.27]	
Without masker	133	573	706
	(107.42)	(598.58)	
	[654.27]	[654.27]	
Column Total	173	964	1137

Table 3 is the table produced for tinnitus with masker occurring in the masker field where cluster 1 represents “moderate to severe hearing loss” and cluster 2 represents “normal or near-normal hearing”. In Table 3, Observed frequencies appear at the top of each cell, Expected frequencies are in ( ), and (Observed frequency – Expected frequency)^2^ values are shown in [ ]. For example, if 40 of the masker fields of the records of patients in cluster 1 contained “masker”, a value of 40 was recorded for masker being associated with that cluster. These values were the “observed” values, denoted O_ij_ in the formula above. The corresponding “expected” values E_ij_ were found by the formula:2

The row total for “tinnitus with masker” is the total number of times “masker” was prescribed to patients in the two clusters = 40 + 391 = 431. The column total for cluster 1 is the total number of patients assigned to cluster 1 over all tinnitus patients = 173. The grand total is the total number of patient records in the study = 1137. Thus the “expected” (in this case E_ij_ = E_11_) number of ‘tinnitus with masker’ in cluster 1 was 431 * 173 / 1137 = 65.58. The significance of this is that the observed value is less than the expected value, suggesting that there is a negative degree of association between the “tinnitus with masker” and the severe hearing loss cluster. The remainder of the test is then performed to discover if this association is statistically significant. Next the O_ij_ and E_ij_ values were used to calculate an overall chi-squared value for the relationship between “tinnitus with masker” and cluster, using the Eq. 1 in Table 4. From this data it could be shown with 99.9% confidence, that these keywords were not randomly distributed, and that some keywords definitely are more associated with some clusters.

**Table 4:** **Overall** ***χ*** ^**2**^

Fields	Overall ***χ*** ^2^	Degrees of freedom (df)	P
Tinnitus with or without masker	18.95	1	P < 0.001
Comments text	4243.87	668	P < 0.001

The words most typical and atypical of each cluster are shown in Tables 5 to 6. These automatically discovered words provided a suitable set of both positive and negative labels for each of the clusters. The labels seem intuitively reasonable. For example, in Table 5, “tinnitus with masker” is found to be atypical of moderate to severe hearing loss cluster, means patients having moderate to severe hearing loss were not using tinnitus masker. This is a common finding in audiology clinics that for severe hearing loss patients, masker might be of no use because they are unable to hear it. In Table 6, it appeared that the patients in cluster 2, the mild to moderate hearing loss group, were more concerned about tinnitus (ringing in the ears) than hearing loss. Thus the words tinnitus and masker (a device using the sound to provide an immediate sense of palliative relief from tinnitus) were typical of this cluster and also are atypical of cluster 1, the severe hearing loss group. Similarly, in (Table 6) cluster 1, the atypical words “canc” (cancelled) and “dna” (did not attend) show that patients with severe hearing loss were less likely to cancel (or simply fail to attend) their appointments. “Tinnitus” appears as “tinnitu” and “Suitable” appears as “suitabl” in Table 6, since all the text was passed through Porter’s (Porter 1980) stemmer for the removal of grammatical endings.

**Table 5:** **Clusters for the records with tinnitus with or without masker field with positive and negative keywords**

	Positive keywords	Negative keywords
C1	Not found	Tinnitus with masker
C2	Not found	Not found

**Table 6:** **Clusters for the records with text field (comments text) with positive and negative keywords**

	Positive keywords	Negative keywords
C1	audio, mould, be34, be52, be36, unmask, be54, sil, ref, tsa, gp, ca, OTHERS, rt, suitabl, be201	masker, rev, tinnitu, appt, fta, help, review, aid, further, nfa, progress, 2000, ok, canc, counsel, cope, 2001, dna
C2	masker, rev, tinnitu, appt, fta, help, review, aid, further, nfa	audio, mould, be34, be52, be36, unmask, be54, sil, ref

### Chi-squared and associations in tabular audiology data for tinnitus masker

The Chi-squared test can show the existence of associations in the data, and other association measures based on the contingency table can be used to measure the strength of the relationship between the variables in medical data. Support and confidence are measures of the interestingness of associations between variables (Ordonez et al. 2006; Bramer 2007). They show the usefulness and certainty of discovered associations. Strong associations are not always interesting, because support and confidence do not filter out uninteresting associations (Han and Kamber 2006). Thus, support and confidence in the medical domain should be augmented by chi-squared (*χ*^2^).

### Discovery of associations with the Chi-squared test tables

The associations of each of the variables (age, comments text, gender, ITE/BTE aid and mould) with “tinnitus with masker” are shown in Table 7 with their overall chi-squared values. In Table 8, typical and atypical keywords found for tinnitus masker associated with variables mentioned above are given, where *, **, *** and **** denote a “tinnitus masker”, “comments text”, “ITE/BTE hearing aid” and “mould” categories respectively. For example, in Table 8 keywords “tinnitu” for “tinnitus” and “counsel” were found associated with tinnitus without masker that is, tinnitus suffering patients who are NOT using masker were counselled on tinnitus. Mould “N8” was found associated with tinnitus masker and mould “2107” for people with tinnitus but not using masker. It is important to mention that BTE aids were found atypical with tinnitus masker.

**Table 7:** **Overall** ***χ*** ^**2**^ **with tinnitus with/without masker**

Fields	Overall ***χ*** ^2^	Degrees of freedom (df)	P
Age	11.31	1	P < 0.001
Comments text	416.49	83	P < 0.001
Gender	0.02	1	P = 0.8875
BTE/ITE aid	17.16	1	P < 0.001
Mould	93.16	2	P < 0.001

**Table 8:** **Categories with positive and negative keywords in records with a tinnitus with/without masker**

	Positive keywords	Negative keywords
Age < =55	*	*
	Not found	Not found
Age > 55	*	*
	Not found	Not found
Tinnitus without masker	**	**
	counsel, tinnitu	Not found
	***	***
	Not found	Not found
	****	****
	2107	N8
Tinnitus with masker	**	**
	masker	counsel, tinnitu
	***	***
	Not found	BTE
	****	****
	N8	2107
Male	*	*
	Not found	Not found
Female	*	*
	Not found	Not found

An audiologist from a large NHS facility said that the “N8” mould is an open fitting ear mould which doesn’t obstruct the ear canal, so allows natural hearing to complement output from the hearing aid and is required when there is a small or no hearing loss, for example, in tinnitus maskers. Patients with significant hearing loss with tinnitus are generally given hearing aids and the environmental sounds picked up will mask the tinnitus. Hence there is a high correlation between “N8” mould and tinnitus masker.

### Measures of association (Yule’s Q) in categorical audiology data for tinnitus masker

The Chi-squared test informed about the presence of an association, while Yule’s Q produces the strength and direction of the association. Yule’s Q is in the range -1 to +1, where the sign indicates the direction of the relationship and the absolute value indicates the strength of the relationship. In Tables 9, 10, 11, 12, Yule’s Q values for tinnitus with/without masker and severe hearing loss cluster/mild-to-moderate hearing loss cluster, hearing aid type, age and gender are given. The symbols “(P)” and “(A)” in tables, stand for present and absent. In Table 10, a Yule’s Q value of 0.57 shows that there is a positive association between the ITE aid and tinnitus with masker, which could be related with the results found in Table 8 above, that BTE aids were atypical of tinnitus with masker. In Table 11, there was a weak positive association of “tinnitus with masker” with Age < =55, meaning that patients of Age < =55 having tinnitus tended to use a masker, while patients of Age > 55 having tinnitus tended not to use a masker.

**Table 9:** **Yule’s Q for tinnitus with/without masker and mild-to-moderate hearing loss cluster/normal or near-normal hearing loss cluster**

Tinnitus	Severe hearing loss cluster (P)	Normal or near-normal hearing loss cluster (P)	Severe hearing loss cluster (A)	Normal or near-normal hearing loss cluster (A)	Yule’s Q
With masker	40	391	133	573	−0.39
Without masker	133	573	40	391	0.39

**Table 10:** **Yule’s Q for ITE/BTE aid and tinnitus with/without masker**

Hearing aid type	With masker (P)	Without masker (P)	With masker (A)	Without masker (A)	Yule’s Q
ITE	67	101	15	82	0.57
BTE	15	82	67	101	−0.57

**Table 11:** **Yule’s Q for tinnitus with/without masker and age**

Tinnitus	Age < =55 (P)	Age > 55 (P)	Age < =55 (A)	Age > 55 (A)	Yule’s Q
With masker	240	182	328	377	0.20
Without masker	328	377	240	182	−0.20

**Table 12:** **Yule’s Q for tinnitus with/without masker and gender**

Tinnitus	Male (P)	Female (P)	Male (A)	Female (A)	Yule’s Q
With masker	246	206	396	337	0.01
Without masker	396	337	246	206	−0.01

### Support and confidence for tinnitus masker associations

Support and confidence are measures of the interestingness of a rule. Support reflects the usefulness of a rule, and confidence its certainty (Han and Kamber 2006). To find the significant associations, the criteria that support is more than or equal to a minimum threshold values called minsup, which is typically 0.01 (or 1%) and also confidence is more than or equal to a minimum threshold value called minconf, which is typically 0.8 (or 80%) are used (Bramer 2007). So, in Tables 13, 14, 15 the only rule that meet such criteria is tinnitus masker associated with ITE aid in Table 13. The Tables 14 and 15 do not meet this criterion. For example, the overall chi-squared value 17.16 gave 99% confidence that there is indeed an association between tinnitus with/without masker and BTE/ITE aid (as shown in Table 7). The Yule’s Q value of 0.57 for ITE hearing aid and tinnitus with masker (in Table 10) shows that this association is both positive and strong. Rule’s support and confidence values of 0.25 and 0.82 (in Table 13) show this association is only moderately useful, but highly certain.

**Table 13:** **Support and confidence for ITE/BTE aid and tinnitus with/without masker**

	Tinnitus with masker		Tinnitus without masker	
Hearing aid type	Support	Confidence	Support	Confidence
ITE	0.25	0.82	0.38	0.55
BTE	0.06	0.18	0.31	0.45

**Table 14:** **Support and confidence for tinnitus with/without masker and age**

	Age < =55		Age > 55	
Tinnitus	Support	Confidence	Support	Confidence
With masker	0.21	0.42	0.16	0.33
Without masker	0.29	0.58	0.33	0.67

**Table 15:** **Support and confidence for tinnitus with/without masker and gender**

	Male		Female	
Tinnitus	Support	Confidence	Support	Confidence
With masker	0.21	0.38	0.17	0.38
Without masker	0.33	0.62	0.28	0.62

### Conclusion to tinnitus and maskers

In this research the literature survey to find the factors associated with tinnitus and masker is done, and it also uses a number of statistical and data mining techniques for finding the significant factors associated with tinnitus masker. The first technique applied was k-means clustering on audiograms, which found two clusters for “tinnitus with or without masker” and “comments text”. These clusters were also automatically assigned labels that is, keywords associated with each cluster are found, which helps in the interpretation of the cluster. For audiograms k-means clustering method was selected, as it works well with large data and produce quick results. The Chi-squared test is used for the discovery of associations for free text comments, diagnosis, hearing aid type, age and gender. Then the measures of associations for the same factors are calculated using Yule’s Q, support and confidence. In fact, significant associations in the heterogeneous audiology data are looked for, with the ultimate aim of looking for factors which patients would most benefit from being fitted with a tinnitus masker.

The clustering was performed on audiograms which produced two main clusters of audiograms. The associations of clusters of audiograms type with tinnitus were confirmed in the section of clustering, and also by literature review in the “Background and significance” section for tinnitus. Thus, it has been demonstrated that the audiogram is a factor influencing the prescription of a tinnitus masker.

In Table 16, the candidate variables associated with tinnitus masker that is “factors influencing the choice of tinnitus masker” and the reasons for choosing them are summarised. These results demonstrated that tinnitus maskers are more likely to be fit to individuals with milder forms of hearing loss, and the factors age, gender, type of hearing aid and mould were all found significantly associated with tinnitus masker. In particular, those patients having Age < =55 years were more likely to wear a tinnitus masker, as well as those with milder forms of hearing loss. ITE (in the ear) hearing aids were also found associated with tinnitus masker. These variables agree fairly well with those factors found in the literature (as given under the heading of “Tinnitus causes”). Thus, the variables were found in the literature and by using statistical and data mining techniques for tinnitus masker.

**Table 16:** **Candidate variables with their reason for association with research question: factors influencing the choice of tinnitus masker**

Variables found	Reason for choosing the variable(s)
Audiograms	Tinnitus maskers were found atypical with moderate to severe hearing loss cluster of audiograms in Table 5, and Yule’s Q found tinnitus masker associated with normal or near-normal hearing loss cluster of audiograms in Table 9.
Gender	Yule’s Q found very weak positive association of males with tinnitus masker in Table 12.
ITE/BTE aid	Chi-squared test found BTE aids atypical of tinnitus maskers in Table 8. By using Yule’s Q, and Support and Confidence tinnitus maskers were found associated with ITE aids in Table 10 and Table 13 respectively.
Age	Yule’s Q found weak positive association of tinnitus masker with Age < =55 in Table 11.
Mould	Mould was found associated with tinnitus with masker using chi-squared test in Table 8.

A feedback on the results of association of mould with tinnitus masker from a professional audiologist of a large NHS (National Health Services, UK) was also taken to better understand them. The results were obtained with different accuracy for different techniques. For example, the chi-squared test results were obtained with 95% accuracy, for Support and Confidence only those results were retained which had more than 1% Support and 80% Confidence. It is important to mention that some other factors that could not be tested with this data were cosmetic reasons, comfort in wearing, ease of use with spectacles and the measure of masking relief provided by tinnitus maskers.

The sets of variables found in Table 16 will be carried forward for constructing a decision support system (DSS). In future, these variables can be combined into mathematical models which will form the basis of a DSS, where unseen patient records will be presented to the system and the associated probability that the patient should be fitted with a tinnitus masker as opposed to NO tinnitus masker will be returned. By constructing these models, it will be seen which variables contribute most in reaching a final decision for tinnitus masker.

## Study approval

It is a study involving audiology data NOT human directly. This data was given to the Department of Computing, Engineering & Technology, University of Sunderland, Sunderland, UK, for Research purpose by a large National Health Services facility after making the data anonymised. So, there was no need to obtain any permission as long as it is being used for research purpose. The author has been working on this data for his research at University of Sunderland and this paper is one of the outcomes of his research.

## Article summary

### Article focus

- A large database of audiology data from a large NHS (National Health Services, UK) facility
- Data analysis of audiology data using various statistical and data mining techniques
- Tinnitus and masker

### Key messages

- Unconventional study to find factors associated with tinnitus masker
- Association analysis of tinnitus and masker
- Improved quality of service in audiology clinics, so that the variables found can be emphasized, and when the decision support system to predict for tinnitus masker would be developed

### Strengths and limitations of this study

- This research includes both BTE (behind the ear) and ITE (in the ear) hearing aids, as ITE aids are generally not available on NHS (National Health Services) prescriptions
- This research finds variables associated with tinnitus masker
- This study includes comment and feedback given by a professional audiologist from a large NHS facility on the association of mould with tinnitus masker
- It does not include factors such as comfort in wearing of masker, ease of use with spectacles and measure of masking relief
